# A Loop All-Fiber Current Sensor Based on Single-Polarization Single-Mode Couplers

**DOI:** 10.3390/s17112674

**Published:** 2017-11-20

**Authors:** Hao Zhang, Junzhen Jiang, Yu Zhang, Huaixi Chen, Na Zhao, Lingyan Lin, Yishen Qiu

**Affiliations:** 1 Department of Electronic Information Science, Fujian Jiangxia University, Fuzhou 350007, China; zhangyu@fjjxu.edu.cn (Y.Z.); lingyanlin@fjjxu.edu.cn (L.L.); 2 Fujian Provincial Key Laboratory for Photonics Technology, Fujian Normal University, Fuzhou 350007, China; jzjiang@fjnu.edu.cn; 3 Fujian Institute of Research on the Structure of Matter, Chinese Academy of Sciences, Fuzhou 350002, China; hxchen@fjirsm.ac.cn; 4 Academy of OPTO-electronics, Chinese Academy of Science, Beijing 100094, China; zhaona@aoe.ac.cn

**Keywords:** fiber current sensors, single-polarization single-mode, fiber loop, ortho-conjugate retroreflector, system stability

## Abstract

Low current sensitivity and insufficient system stability are two key problems in all-fiber current sensor (AFCS) studies. In order to solve the two problems, a novel AFCS combining single-polarization single-mode (SPSM) couplers and a loop structure is presented in this paper with a design that incorporates the advantages of both SPSM couplers and a loop structure. SPSM couplers are shown to simplify the AFCS system and reduce the risk of interference, and the loop structure can enhance the current sensitivity. Both theory and experiment prove that the new AFCS can simultaneously overcome two prevalent obstacles of low current sensitivity and low stability.

## 1. Introduction

All-fiber current sensors (AFCS) based on the Faraday effect have the potential to replace traditional current transformers due to their advantages like immunity to electromagnetic interference, safety, and light weight over traditional current sensors [1,2]. Many novel designs, such as AFCSs based on magnetic fluid, fiber microwires, and optical switches, have been presented and promote the development of AFCS [3,4,5,6]. However, two obstacles have always prevented their widespread application: low current sensitivity and insufficient system stability [7,8]. These problems result in a small useful signal that is easily obscured by noise.

A simple traditional method of addressing these issues is to use a long fiber to enhance the Faraday effect [9]. However, a long fiber increases the size of the sensor head and makes the system more sensitive to environmental changes. Researchers have tried other solutions to overcome these obstacles, such as using doped fibers to increase the Verdet constant of the system [10], and using spun fibers or flint fibers to reduce the sensitivity to the environmental changes [11,12]. However, those specialty fibers are usually expensive and very difficult to fabricate [6].

Since 2010, some AFCSs based on a repeated configuration in order to increase Faraday effect have been reported [13,14]. An AFCS using a Faraday rotation mirror (FRM) cavity or based on loop architecture, which often has a repeated or cyclic structure, can allow the signal to traverse the sensor head repeatedly and enhance the Faraday effect [14]. However, it is important to note that these configurations use some optical devices that are susceptible to environmental influence, such as fiber couplers and polarization beam splitters (PBS). Therefore, those approaches may compromise the system stability.

As a matter of fact, repeated configuration is a very useful design for AFCS. In order to solve the two problems of low current sensitivity and low system stability, we present a novel loop AFCS based on a single-polarization single-mode (SPSM) coupler. This AFCS use SPSM couplers to replace common couplers and convert the Faraday rotation caused by current into light intensity. The configuration becomes relatively simple and does not require some components, such as PBSs and polarizers, which are common in other AFCSs and may contribute to the system’s sensitivity to interference. As a result, the AFCS not only increases the current sensitivity through its loop architecture, but also improves system stability effectively. Furthermore, this design can also reduce the cost of the system.

## 2. Configuration and Principle

**Figure 1:**
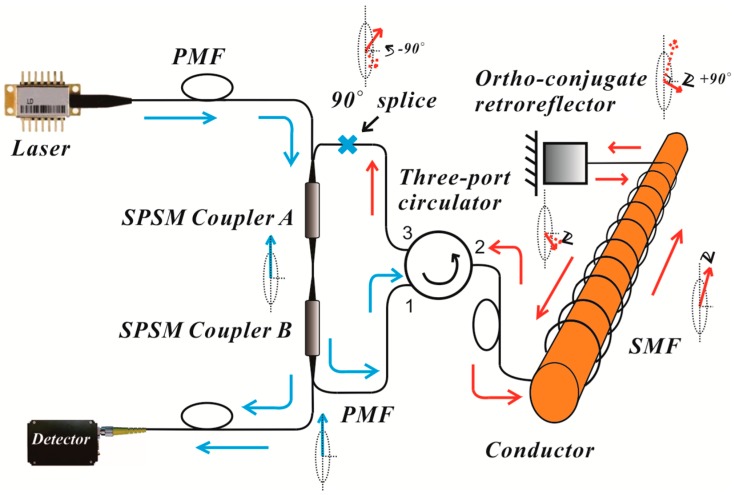
The configuration of the loop AFCS based on SPSM couplers.

**Figure 2:**
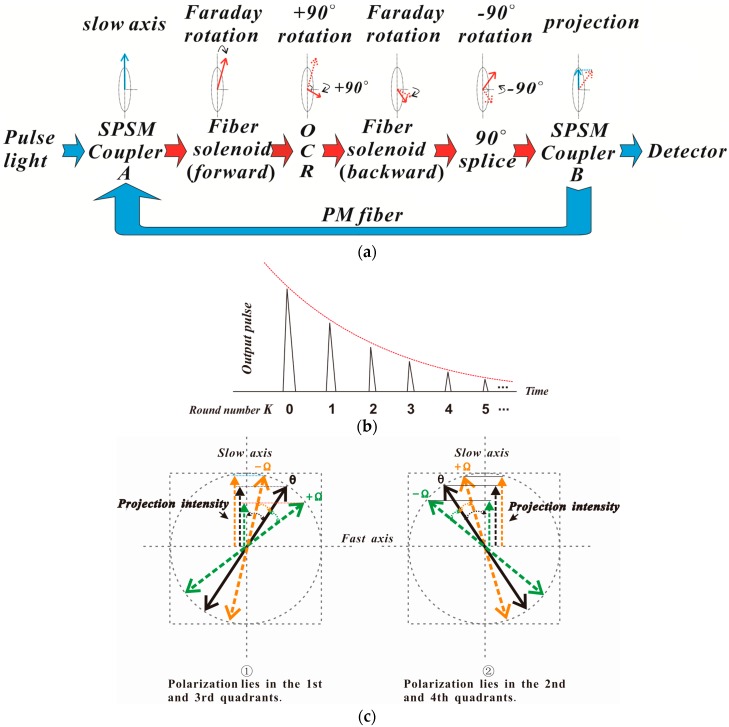
The changing process of light in the loop AFCS based on the SPSM coupler, (**a**) for the change of polarization state of light; (**b**) for the output signal; and (**c**) for the relationship between Faraday rotation angle Ω and the angle *θ*.

Figure 1 shows the configuration of the proposed loop AFCS based on SPSM couplers. The system is comprised of a pulse laser, two SPSM couplers A and B, a three-port circulator, a fiber solenoid, an ortho-conjugate retroreflector (OCR) and a photo-detector. All components are connected along the slow optical axis, and two SPSM couplers and the fiber solenoid constitute the fiber loop structure. The SPSM couplers are employed as both polarizer and analyzer because they only allow light polarized along the slow axis to pass through [15]. The light from the laser is coupled into SPSM coupler A via polarization-maintaining fibers (PMFs) and becomes linearly polarized along the slow axis. The light will circulate inside the loop several times before it is completely attenuated. The polarization state of the light is influenced by the Faraday effect and other effects, such as the birefringence of the fiber as the light traverses the loop structure. The process of polarization alteration is shown in Figure 2a. The polarized light aligned with the slow axis couples to the fiber solenoid via the three-port circulator, reflects at the OCR located at the far end of the fiber solenoid, and passes back through the solenoid in the opposite direction, returning to the loop. As the polarization of the light is rotated 90° by the OCR, the connection between the ports of the three-port circulator and SPSM coupler A is also rotated 90° to ensure that the light passes through the SPSM couplers. The light becomes linearly polarized along the slow axis again when it passes through SPSM coupler B. As shown in Figure 2a, the intensity of light is reduced when it arrives at SPSM coupler B. The effect of the SPSM couplers follows the projection rule (Malus law), and will superpose when the light circulates inside the loop continuously. Therefore, the intensity of the light steadily decreases. In each round trip, a small fraction of the light is coupled into the detector by SPSM coupler B. Finally, the detector receives a series of pulses known as ring-down spectra [16], with the pulse number equal to the round number, as shown in Figure 2b.

The main body of this configuration uses PMF and SPSM couplers, with the exception of the fiber solenoid, to ensure sufficient system stability. The fiber solenoid uses a standard single-mode fiber (SMF) that can improve the anti-interference ability through the OCR which, itself, reduces the influence of birefringence caused by temperature and vibrational fluctuations [17].

Considering that the light attenuation caused by the loss in components and rotation in the loop structure is negatively exponential, the intensity of light output from the fiber loop for each round trip can be described as [9]:

(1) $$J={\left|E_{0}\right|}^{2}\cos^{2}\left(\theta +2\Omega \right)e^{-\alpha },$$

where *E*_0_ is the amplitude of the pulse from the light source, *θ*, caused by the reciprocal rotation and not the Faraday effect, is the angle between the polarization and slow axis. The structural rotation angle, Ω, is the Faraday rotation angle caused by the current, and *α* is attenuation coefficient of the loop structure. For the *K*th round trip, the output light is:

(2) $$J_{K}={\left|E_{0}\right|}^{2}\cos^{2K}\left(\theta +2\Omega \right){\left(e^{-\alpha }\right)}^{K}.$$

This equation shows that the output light varies with the Faraday rotation Ω and the round number *K*. The normalized difference between the output light in the current-on and current-off states can be defined as:

(3) $$\Delta J=\frac{J_{on}-J_{off}}{J_{off}}=\frac{\cos^{2K}\left(\theta +2\Omega \right)-\cos^{2K}\left(\theta \right)}{\cos^{2K}\left(\theta \right)},$$

where *J_off_* is the output light when the current is off, and *J_on_* is the output light when the current is on. As the Faraday rotation Ω is much smaller than the angle *θ* because the Verdet constant of SMF is very small [18], Equation (3) can be expanded and simplified via the binomial theorem. The new expression is given by

(4) $$\Delta J={\left[\frac{\cos\left(\theta +2\Omega \right)}{\cos\left(\theta \right)}\right]}^{2K}-1\approx -4K\Omega \tan\left(\theta \right)=-4KVNI\tan\left(\theta \right).$$

where *V* is the Verdet constant of the fiber, and *N* is the number of turns of the fiber solenoid. Equation (4) shows that the normalized difference of output light is directly proportional to the round number *K* and current *I* in the approximation condition. Therefore, the current can be measured via the normalized difference of output light △*J*, and the current sensitivity coefficient *k_current_* is:

(5) $$k_{current}=\left|4KVN\tan\left(\theta \right)\right|.$$

The current sensitivity obviously increases with *K* and *N*. The maximum measurable current of the configuration depends on the quadrant of polarization of signal, the angle *θ*, and the direction of Faraday rotation Ω, as shown in Figure 2c. This is given by:

(6) $$I_{\max}=\{\begin{array}{c} \frac{\theta }{2KVN}\;1\mathrm{st}\;\mathrm{and}\;3\mathrm{rd}\;\mathrm{quadrants}\;\left(-\Omega \right)/2\mathrm{nd}\;\mathrm{and}\;4\mathrm{th}\;\mathrm{quadrants}\;\left(+\Omega \right)\\ \frac{\pi /2-\theta }{2KVN}\;1\mathrm{st}\;\mathrm{and}\;3\mathrm{rd}\;\mathrm{quadrants}\;\left(+\Omega \right)/2\mathrm{nd}\;\mathrm{and}\;4\mathrm{th}\;\mathrm{quadrants}\;\left(-\Omega \right) \end{array}.$$

Figure 2c shows that the angle between the polarization axis and the slow axis is *θ* when current was switched off. For the polarization lying in the first and third quadrants, the angle is *θ* + Ω when Ω changes in a clockwise direction with current rise, and *θ* − Ω when Ω changes in an anticlockwise direction. For the polarization lying in the second and fourth quadrants, the opposite is true. The change of polarization cannot go beyond one quadrant. Therefore, the maximum allowed Faraday rotation Ω is equal to *θ* when the polarization lies in the first and third quadrants and Ω changes in an anticlockwise direction, or when the polarization is in the second and fourth quadrants and Ω changes in a clockwise direction. Similarly, the maximum allowed Faraday rotation Ω is *π*/2 − *θ* when polarization lies in the first and third quadrants and Ω changes in a clockwise direction, or when the polarization lies in the second and fourth quadrants and Ω changes in an anticlockwise direction. The maximum measurable current can be calculated given *θ*, *V*, *N*, and *K*. For example, for a system with 100 turns of the fiber solenoid, whose Verdet constant is 0.25 μrad/A at 1550 nm, and *θ* is *π*/4, the maximum measurable current is 15,708 A for *K* = 17,854 A for *K* = 2, and 5236 A for *K* = 3.

## 3. Experiments and Discussion

**Figure 3:**
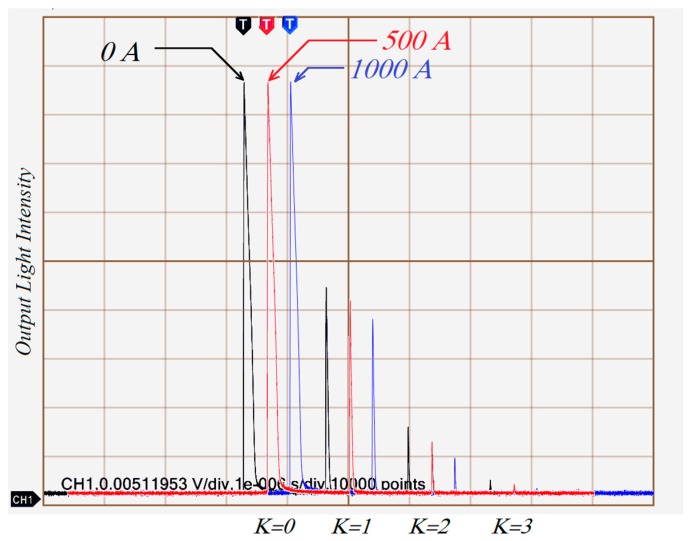
Intensity variations of output light for currents of 0 A, 500 A, and 1000 A passing through the sensor head. Measurements were carried out with a Tektronix TDS3054B 500 digital oscilloscope.

The experimental layout of the loop AFCS based on the SPSM couplers shown in Figure 1, was comprised of a 1550 nm pulsed laser diode (LD, model MS3400-1550, average output power 3.8 mW, peak power 10 W, pulse width 15 ns, output frequency 10 kHz, Connet Co., Ltd., Shanghai, China), two 2 × 1 fiber SPSM couplers (both split ratios 5%:95%), a three-port circulator, a fiber solenoid with 200 turns, and a photo-detector (DET01CFC, peak response R*_λ_* = 0.95 A/W at 1550 nm, Thorlabs Co., Newton, NJ, USA). The output from photo-detector was displayed by a digital oscilloscope (TDS3054B, Tektronix, Beaverton, OR, USA). In the experiment, the SPSM couplers and the three-port circulator were protected by a heat-insulating cavity filled with fiberglass in order to improve the stability of the system. Firstly, the current response of the AFCS was tested. The direct current ranged from 0 to 1200 A. The intensity variations of output light when 0 A, 500 A, or 1000 A currents were passed through the fiber solenoid are shown in Figure 3. 

**Figure 4:**
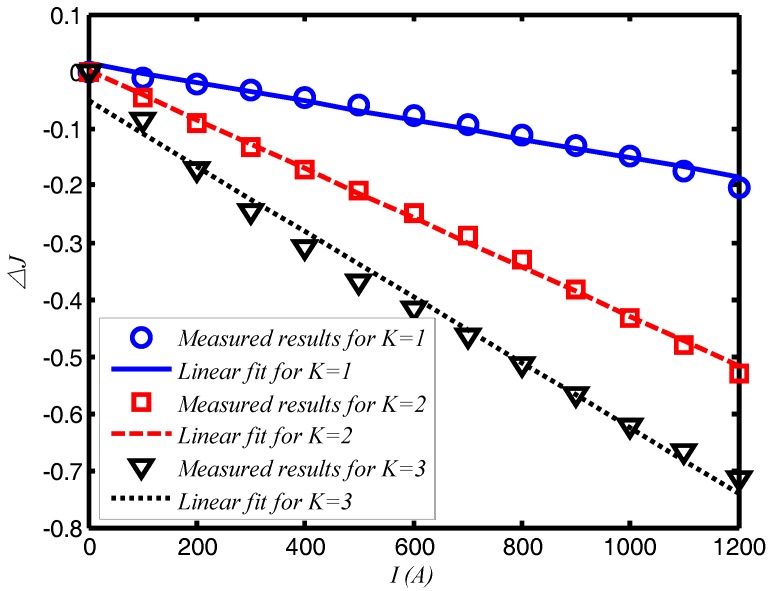
The relationship between current and the normalized output light difference △*J*. The linear fits are based on the least squares method.

There are three series of output light data in Figure 3. The black represents the output for 0 A, the red for 500 A, and the blue for 1000 A. In this experiment, the angle *θ* between the polarization axis and the slow axis was measured when current was switched off, which yielded a value of *π*/4. The Faraday rotation Ω acts in a clockwise direction. Figure 3 shows that each series of output light data has four peaks with *K* ranging discretely from 0 to 3, and these peaks vary with current and round number *K*. According to Equation (6), the maximum measurable current is 7854 A for *K* = 1, 3927 A for *K* = 2, and 2618 A for *K* = 3. The relationship between current intensity and output light is shown in Figure 4.

Figure 4 shows a strong linear relationship between the current intensity and the normalized output light difference △*J*. This observation agrees with Equation (4). The gradients of the curves in the figure represent the current sensitivity, which gradually increases with the round number *K*. For *K* = 1, the current sensitivity is approximately 1.67 × 10^−4^/A; for *K* = 2, the sensitivity is about 4.25 × 10^−4^/A; and for *K* = 3, it is 6.17 × 10^−4^/A. This means that the current sensitivity when *K* = 2 is about 2.5 times larger than that for *K* = 1, and the value for *K* = 3 is 3.7 times larger than that for *K* = 1. Therefore, the system can operate at different current sensitivities by selecting different values of *K*s. In this experiment, the output light intensity is very low when *K* = 3 and is highly susceptible to noise. Thus, the *K* = 3 setting is inappropriate for actual measurements. Although the round number does not take very large values, the current sensitivity of loop AFCSs using SPSM couplers can increase to at least four times that of basic AFCSs using single polarization detection [5], because it doubles the optical path of the light signal via the OCR component.

**Figure 5:**
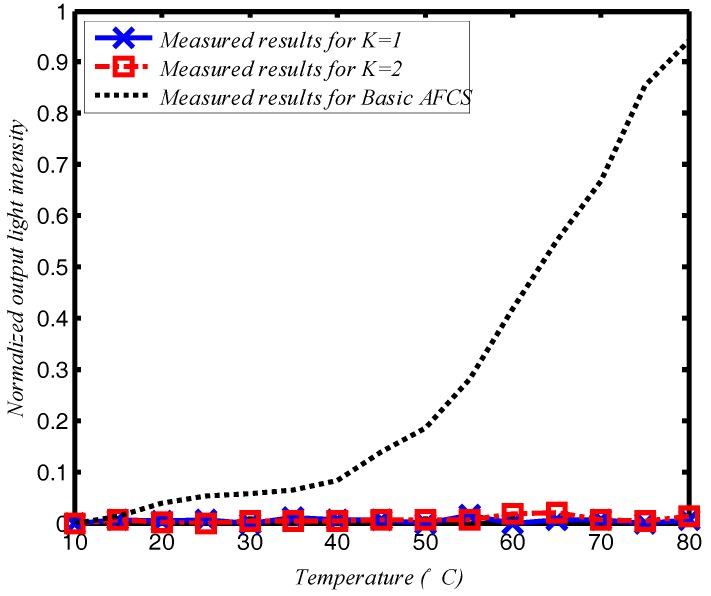
The effect of temperature on the loop AFCS based on the SPSM couplers.

The temperature and vibrational stability of the system was also tested. In the temperature test, the entire AFCS, excluding the light source and photo-detector, was placed in a temperature-controlled cabinet. The experiment was performed for temperatures ranging from 10 °C to 80 °C. The results are read two minutes after the temperature is stable and recorded every one degree. For comparison, a basic polarimetric AFCS that used the same fiber solenoid with 200 turns as a sensor head was also tested under the same conditions. Figure 5 shows the relationship between the temperature and normalized output light intensity. The normalized output light intensity in the figure can be given by (*J_M_* − *J*_0_)/*J*_0_, where *J_M_* is measured light intensity, and *J_0_* is output light when AFCS at room temperature of 20 °C.

The basic AFCS design is highly sensitive to temperature changes, as shown in Figure 5. Therefore, basic AFCSs without any compensatory or control setting are ineffective in real environments, whereas temperature has little effect on the loop AFCS using SPSM couplers for both *K* = 1 and *K* = 2. In this experiment, the temperature influence on the loop AFCS using SPSM couplers is about 17 dB less than that for the basic AFCS design.

**Figure 6:**
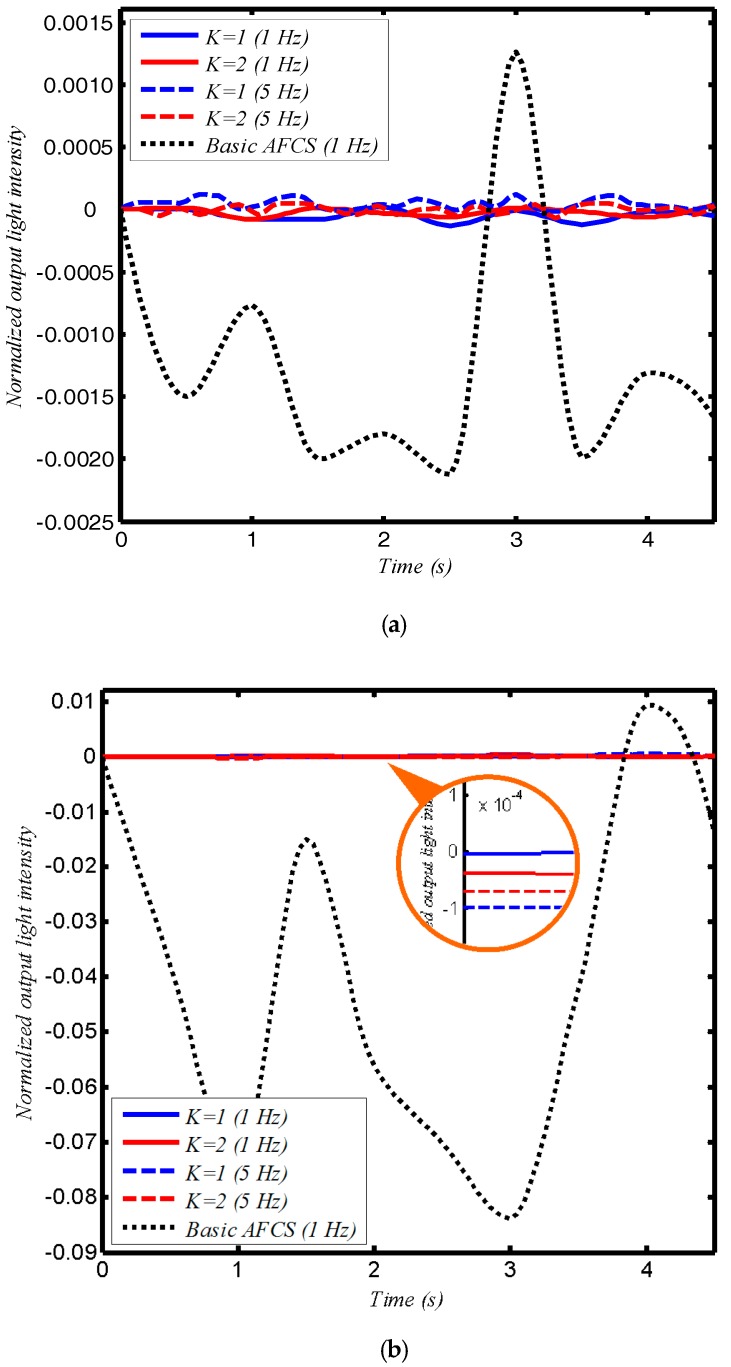
The effect of vibration on the loop AFCS based on the SPSM couplers, (**a**) for 1 mm amplitude, and (**b**) for 10 mm amplitude.

In the vibration test, the configurations were placed on a vibrating platform. Two vibrational frequencies were chosen, 1 Hz and 5 Hz with amplitudes of 1 mm and 10 mm used for both frequencies. As with the temperature test, this experiment also tested the basic AFCS design. The results are shown in Figure 6. The normalized output light intensity in Figure 6 is also (*J_M_* − *J*_0_)/*J*_0_, and *J_0_* is the output light when AFCS is free from vibrational interference.

The results show that the loop AFCS using SPSM couplers is more robust than the basic design under external vibrations. Altering the vibrational frequency and amplitude has no obvious effect on the output light intensity of the loop AFCS using SPSM couplers, but causes large intensity changes in the basic AFCS design. Here, we defined the time-average of the absolute normalized output light intensity of the AFCS under external vibration as the influence of vibration on the AFCS. In the experiment, the influence of vibration on the configuration using SPSM couplers could be reduced by 10 dB compared to the basic design when the vibration amplitude was 1 mm, and reduced by more than 20 dB when the vibrational amplitude was 10 mm. This suggests that the new system reduces the influence of large vibrations more effectively than smaller vibrations.

## 4. Conclusions

In summary, a novel AFCS combining the SPSM couplers and the loop configuration is presented in this paper. It not only significantly reduces the effect of environmental influences such as temperature and external vibration, but also has advantages in current sensitivity and structural simplification. The experiments prove the advantages and the feasibility of the AFCS design. This allows us to develop a new loop AFCS, which gets us close to practical use.
